# Autoantibodies in the follow-up of autoimmune hepatitis

**DOI:** 10.1016/j.jtauto.2026.100369

**Published:** 2026-04-01

**Authors:** Gábor Nagy, Dóra Bencze, Sarolta Demeter, Krisztina Pénzes-Daku, Lilla Szabó, Beáta Tóth, Róza Földesi, Mária Papp, Péter Antal-Szalmás

**Affiliations:** a Department of Laboratory Medicine, Faculty of Medicine, University of Debrecen, Debrecen, Hungary; b Department of Gastroenterology, Faculty of Medicine, University of Debrecen, Debrecen, Hungary; c Doctoral School of Medical Sciences, University of Debrecen, Debrecen, Hungary

**Keywords:** Autoimmune liver disease, Autoimmune hepatitis, Antinuclear antibodies (ANA), Smooth muscle antibodies (SMA), Anti–liver kidney microsomal type 1 antibodies (anti-LKM1), Anti–soluble liver antigen/liver pancreas antibodies (anti-SLA/LP)

## Abstract

Autoimmune hepatitis (AIH) is a rare, chronic, immune mediated liver disorder with unknown origin, associated with elevation of serum transaminases and IgG, positivity of certain autoantibodies and characteristic histopathological alterations in the liver. The clinical scenario is variable, the disease can be asymptomatic for a long time, noticed accidently by liver enzyme elevation or diagnosed only when liver cirrhosis has already developed. In rare cases acute hepatitis with liver failure is the starting event. In general, immunosuppressive treatment is required to diminish the progression of liver destruction and to prevent liver transplantation. The most important autoantibodies involved in AIH diagnostics – antinuclear antibodies, smooth muscle antibodies, anti–liver kidney microsomal type 1, anti-liver cytosolic antigen 1 or anti–soluble liver antigen – can be used for definition of AIH subtypes, too. These parameters are included in current AIH diagnostic guidelines. Since the fate of the patients depends very much on the proper choice and efficacy of the applied therapy prognostic and predictive markers of disease outcome can be essential in initiating therapy, while markers of disease activity and therapy response can support follow-up. Some biochemical parameters or histopathological data can be used for these purposes, but the potential role of AIH-specific autoantibodies in these is not entirely clear. In this current review the potential use of classical and emerging autoantibodies in diagnostics, prognostics, and follow-up of AIH patients will be discussed.

## 1 Introduction

Autoimmune hepatitis (AIH) is a chronic, immune-mediated inflammatory liver disease characterized by a loss of tolerance to hepatocyte-specific antigens, leading to persistent immune attack against liver tissue. Despite international endeavor to reveal pathophysiology, the exact disease processes remain unclear. The pathogenesis involves a complex interplay of genetic predisposition, such as associations with certain HLA haplotypes (e.g., HLA-DR3, HLA-DR4), and environmental triggers, including infections or drugs, which can initiate autoreactive T-cell activation. This results in ongoing hepatocellular injury, interface hepatitis on histology, and progressive fibrosis if untreated [[1], [2], [3], [4]]. Autoimmunity has a crucial role in the development of AIH as signaled by the presence of autoantibodies and elevated immunoglobulin levels in the majority of cases [1,2,5,6].

The incidence of AIH varies geographically, but it is estimated at 1–2 cases per 100,000 population annually, with a prevalence of approximately 10–25 cases per 100,000. It predominantly affects females, accounting for around 70–80% of cases, and although it may occur at any age, peaks are observed in adolescence and middle adulthood. AIH is considered rare, but it represents an important cause of chronic liver disease that may progress to cirrhosis and liver failure without timely intervention [2,7,8].

Clinical manifestations are diverse, ranging from asymptomatic cases detected incidentally by elevated liver enzymes to acute severe hepatitis with jaundice or even acute liver failure. The most common form of presentation is chronic hepatitis, with symptoms including fatigue, malaise, abdominal discomfort, loss of appetite, weight loss, muscle aches, jaundice and joint pain. Approximately 25-35% of patients already have cirrhosis when the diagnosis is made, which can be asymptomatic or causes ascites, splenomegaly and cutaneous stigmata (caput medusae - dilated abdominal wall veins, spider angiomas). Laboratory findings typically show elevated aminotransferases, hypergammaglobulinemia and the presence of autoantibodies. Liver histology is also an important component of the diagnostic work-up. The clinical course is variable, but early diagnosis and immunosuppressive therapy are crucial to prevent irreversible liver damage [[9], [10], [11], [12], [13]]. Due to lack of sensitive and specific biomarkers, diagnosis still needs complex evaluation of clinical symptoms, liver histology, imaging data and laboratory tests. Autoantibodies are an inevitable part of the diagnostic work-up but less important during follow-up [[9], [10], [11]]. This paper focuses on this less characterized role of autoantibodies in monitoring of autoimmune hepatitis.

## 2 Autoantibodies in autoimmune hepatitis

### 2.1 Methods and standardization

**Fig. 1:**
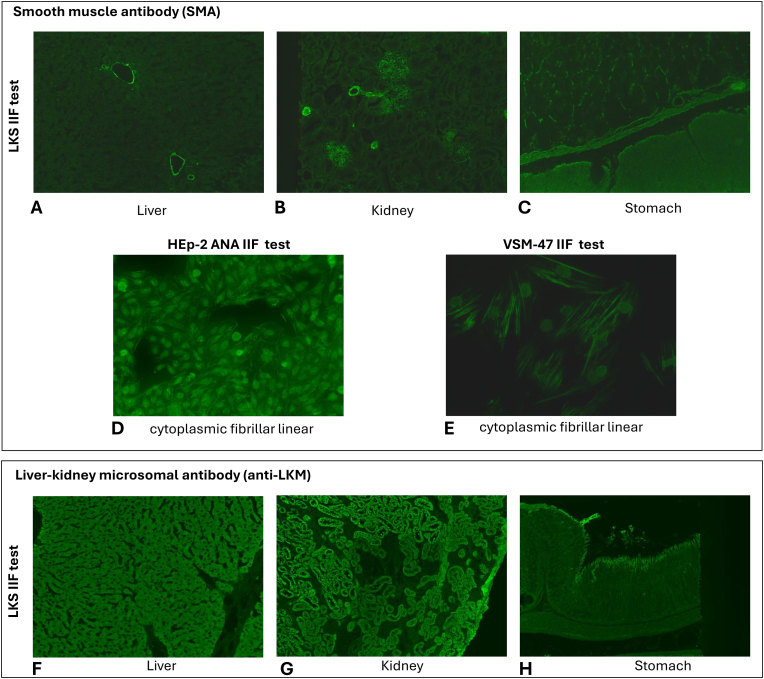
Fluorescence patterns observed in the presence of smooth muscle antibody and anti-liver-kidney microsomal antibody using indirect immunofluorescence methods. SMA can be seen on rodent liver/kidney/stomach sections (A,B,C), but also on HEp-2 (D) and vascular smooth muscle (VSM-47) cell (E) layers. Anti-LKM antibodies cause very fine speckled cytoplasmic staining of hepatocytes (F) and proximal tubular cells (G), but stomach section is negative (H).

Multiple autoantigens associated with autoimmune liver diseases were discovered in the past decades using mostly indirect immunofluorescence assay (IFA) as autoantibody detection test. The substrate is usually a complex block of rat or mouse liver, kidney and stomach tissue sections which constitutes the ‘LKS’ or ‘triple tissues’ test, accepted as a good screening test, despite missing some autoantibodies due to differences between human and rodent antigens. Monkey tissues would be better also because of lower background and heterophile staining but rodent tissues are more easily accessible. Rodent LKS assays can identify anti-smooth muscle (SMA), anti-liver-kidney microsome (anti-LKM) and anti-liver cytosol 1 (anti-LC1) autoantibodies (Fig. 1) involved in the diagnostics of AIH, in this way making it an excellent screening test for AIH [14].

The outcome of the LKS assay might depend a lot on the manufacturers, but the proper evaluation of the tissue elements showing positive reaction requires deep knowledge and great experience. One of the most important autoantibodies in diagnostics of AIH is smooth muscle specific antibody (SMA). Six different anatomical part of the LKS fields can give a positive reaction in the presence of these antibodies: i) muscle elements of the tunica muscularis and lamina muscularis mucosae in stomach; ii) interglandular fibers in stomach; iii) vascular cell wall elements in liver; iv) vascular cell wall elements in kidney; v) mesangial cells of glomeruli in kidney; vi) fibers surrounding the tubules in kidney (Fig. 1A,B,C). The exact definition of the tissue elements to be involved in SMA positivity evaluation is still missing. A recent study testing 61 AIH sera and 72 nonalcoholic fatty liver disease controls could show that there was a clear difference in the analytical performance of the LKS IIF test depending on which tissue elements were taken account. The highest diagnostic specificity and positive predictive value for AIH was achieved if vascular, glomerular and tubular reactivity was present together in a patient sample [15]. Other IIF assays can help the identification of SMA, like the HEp-2 cells used for detection of ANA. The cytoplasmic fibrillar linear (AC-15) pattern shows the presence of actin specific antibodies with a high specificity [15] (Fig. 1D). Furthermore, a special immortalized vascular smooth muscle cell line (VSM-47) can help the identification of actin-specific antibodies showing similar IIF pattern that can be observed in HEp-2 cells [15] (Fig. 1E).

ANA HEp-2 tests can help not only identification of anti-actin antibodies in the cytoplasm of the cells, but a positive reaction can be noticed in the nuclear area of the cells, too. In AIH we see most frequently a homogenous pattern (AC-1), but the exact antigenicity of this reaction is not well known. Another analytical problem in AIH diagnostics is the proper substrate used for ANA detection. Traditionally ANA positivity was judged using the rodent LKS tissues, namely the nucleus of the hepatocytes was evaluated. The suggested screening sample dilution was 1:40 in this peculiar assay and positivity at this and at the 1:80 dilution was embedded into diagnostic guidelines. Recently, HEp-2 cell-based tests became wide-spread for ANA evaluation because of their large and structured nucleus that makes evaluation much easier. Furthermore HEp-2 cells are proliferating that makes possible the identification of the cell-cycle dependent autoantibodies, too. The problem is that serum samples of healthy individuals at 1:40 dilution give higher rate of – false – positivity on HEp-2 cells, and because of this a 1:80 or 1:160 dilution is suggested for screening in these tests, making impossible their incorporation into the calculation of AIH diagnostic score values. Recent publications suggest equivalence between a 1:40 positivity on rodent tissue and 1:80-1:160 on HEp-2 cells and between a 1:80 positivity on rodent tissue and 1:160-1:320 on HEp-2 cells, that has to be determined by each laboratory for its own assays [10,15].

Antibodies to identified autoantigens can be determined using antigen specific solid phase tests, such as ELISA, fluorescence or luminescence immunoassay and immunoblot. These have the advantage of being more specific and sensitive than the recognition of the corresponding pattern of the IIF test. Furthermore, the immunoblot technique enables multiplex testing, which is useful with regard to the number of possible autoantibodies in a patient suspected to have autoimmune liver disease. The disadvantage of the immunoblot technique compared to ELISAs is that it provides qualitative or semiquantitative data, and it has moderate analytical sensitivity compared to the highly sensitive and quantitative ELISAs. Results of indirect immunofluorescence and immunoblot tests are used as qualitative tests in some studies, which can mask a significant change in antibody level within the positive range. Semi-quantitative assays, and especially quantitative assays, are more appropriate for follow-up, ELISA tests could improve the utility of autoantibodies as activity markers, although they are less common or not available at all for some antibodies. The spread of fluorescence or luminescence immunoassays may lead to further improvement in the follow-up of antibodies, because the wider range of measurement signals makes them more precise in an analytical sense.

Standardization of autoantibody testing is difficult as a consequence of the heterogeneity of the antibodies targeting different epitopes on the same autoantigen [16]. In case of autoimmune liver diseases, the use of different methods or reagents further increases variability of results [17]. Some guidelines are available, but harmonization should be continued [18]. The most critical assay component is the antigen; source of antigen determines the autoantibodies that can be detected by a test and is also a key factor in diagnostic sensitivity and specificity. The number of antibodies detected and their quantitation depend on the type of method. Certain assay forms can identify PBC specific autoantibodies, which is an important feature, since signs and symptoms of AIH and PBC may be similar and overlap syndrome is frequent. IIF tests are suitable to detect antibodies against unknown/uncharacterized antigens. Comparison of autoantibody testing methods used in the diagnostics of AIH are shown in Table 1.

**Table 1:** Comparison of methods used for detection of most relevant autoimmune hepatitis specific or related autoantibodies.

Method	Indirect immunofluorescence test on HEp-2 cells	IIF - LKS tissues	Immunoblot/ line immunoassay	Enzyme/luminescence/fluorescence immunoassay (ELISA, LIA, FEIA)
Antibody	- chromatin - nuclear homogeneous (AC-1): - anti-dsDNA, anti-histone, anti-nucleosome, other antibodies - cytoskeleton - actin-like (AC-15): - anti-F-actin	- ANA - SMA - anti-LKM - anti-LC	- anti-F-actin - anti-SLA/LP - anti-LKM-1 - anti-LC-1 - anti-Ro52	- anti-F-actin - anti-SLA/LP - anti-LKM-1 - anti-LC-1 - anti-Ro52
Analytical sensitivity	low	medium	medium	high
Analytical specificity	low	low	medium	medium
Antigen source	human epithelial cell layer	rat/mouse tissue	purified animal or recombinant human	purified animal or recombinant human
Number of antibodies detected	multiple	multiple	multiplex	single
Quantitation	qualitative or semiquantitative (titer)	qualitative or semiquantitative (titer)	qualitative or semiquantitative (negative, +, ++, +++)	quantitative (arbitrary unit)
Standardization of method	International Consensus on ANA Patterns (ICAP)	-	-	-
International control sample	IS2072 ANA Homogeneous/rim pattern (AC-1)	-	-	-
Which primary biliary cholangitis related antibody/pattern can be detected?∗	- multiple nuclear dots (AC-6) - punctate nuclear envelope (AC-12) - centromere (AC-3) - mitochondria-like/cytoplasmic reticular (AC-21)	- AMA	depends on reagent	none
Can unknown/uncharacterized antibodies be detected?	yes	yes	no	no

AMA: anti-mitochondrial antibody; ANA: antinuclear antibody; IIF: indirect immunofluorescence; LC: liver cytosol; LKM: liver-kidney microsomal; LKS: rodent liver, kidney, stomach; SLA/LP: soluble liver antigen/liver pancreas; SMA: smooth muscle antibody. Homepage of ICAP: https://www.anapatterns.org. International control sample is available from: https://www.plasmaservicesgroup.com/iuis-ana-standards. ∗: useful in differential diagnostics and in case of overlap syndromes.

Patient population has a huge impact on autoantibody data, individuals from different countries, geographical areas and races can produce various antibodies in an autoimmune disease. Prevalence of autoantibodies in AIH is heterogeneous, patient population was indicated as a factor causing variability of sensitivity in a meta-analysis [19].

### 2.2 Diagnostic autoantibodies in autoimmune hepatitis

#### 2.2.1 Autoantibodies involved in scoring systems

Diagnosis of autoimmune hepatitis is based on complex assessment of clinical, histology and laboratory data. Importance of autoantibodies is reflected by their incorporation into the original (1993), the revised (1999) and the simplified (2008) International Autoimmune Hepatitis Group (IAIHG) scoring systems [[20], [21], [22]].

The core autoantibodies incorporated in all systems are antinuclear antibodies (ANA), smooth muscle antibodies (SMA), and anti–liver kidney microsomal type 1 antibodies (anti-LKM1). ANA and SMA are the hallmark markers of type 1 AIH, present in approximately 60–80% of patients, and their diagnostic relevance is strengthened at higher titers (≥1:40 in adults). Anti-LKM1 is characteristic of type 2 AIH, occurring predominantly in children and adolescents, and is considered highly specific for the disease.

Other autoantibodies are included in the original and the revised criteria systems but scored only in patients negative for ANA, SMA and LKM-1. The anti–soluble liver antigen/liver pancreas antibody (anti-SLA/LP), although less frequently detected, is highly specific for AIH and may be the sole serological marker in some patients. It is associated with a more severe disease course and higher relapse rates. This antibody was formally included in the 2008 simplified criteria (at that time it was known that anti-SLA and anti-LP target the same protein). Anti–liver cytosol type 1 antibody (anti-LC1), often present in conjunction with anti-LKM1, contributes to subtype definition in pediatric and atypical cases. Atypical perinuclear anti-neutrophil cytoplasmic antibodies (p-ANCA) are frequently observed in AIH, primary sclerosing cholangitis (PSC) and inflammatory bowel disease, but lack of specificity limits their diagnostic weight. Anti-asialoglycoprotein receptor (ASGPR) antibodies are the only organ specific reactivities in autoimmune hepatitis and may have a pathogenic role by targeting hepatocyte surface receptors. Prevalence is high (67-88%) in AIH, though a recent study reported lower sensitivity and similar prevalence (33%) in PSC [23,24].

The scoring systems also address non-AIH autoantibodies to enhance diagnostic accuracy. Anti-mitochondrial antibodies (AMA), characteristic of primary biliary cholangitis (PBC), were assigned negative points in the 1993 and 1999 systems to reduce misclassification, while the 2008 system emphasizes their role in identifying AIH–PBC overlap syndromes rather than assigning a score [[20], [21], [22]].

#### 2.2.2 Other antibodies not included in current scoring systems

Anti-LKM2 antibodies are directed against cytochrome P450 2C9 (CYP2C9), occurring rarely in drug-induced autoimmune-like hepatitis (e.g., tienilic acid–associated hepatitis) rather than idiopathic AIH [25]. Anti-LKM3 antibodies target uridine diphosphate glucuronosyltransferase (UGT1A) and are found in some patients with AIH type 2 and hepatitis D virus infection, serving as a useful but rare serological marker [26].

Anti-Ro52/TRIM21 antibodies occur in 25-40% of patients with AIH (often in combination with anti-SLA) but the diagnostic specificity is low due to their appearance in other autoimmune diseases including Sjögren's syndrome, systemic lupus erythematosus, systemic sclerosis and idiopathic inflammatory myositis [[27], [28], [29]].

Patients with AIH had significantly higher levels of antibodies to cytokeratin 8, 18, and 19 than healthy volunteers or patients with chronic active hepatitis C. Furthermore, levels decreased following steroid treatment [30]. Anti-α-actinin antibodies target the actin binding cytoskeletal protein α-actinin and have been identified in autoimmune hepatitis type 1 and are present in two thirds of patients with anti-filamentous actin (anti-F actin) positivity [31]. Anti-beta-tubulin 5 antibodies were reported in 68% of untreated autoimmune hepatitis patients but also in PSC and other autoimmune diseases, levels decreased under immunosuppressive therapy [32].

Anti-glutathione S-transferase T1 (anti-GSTT1) antibodies target the hepatocyte cytosolic enzyme and were found in de novo AIH after liver transplantation as a consequence of alloreactive immune response [33].

Anti-*Saccharomyces cerevisiae* antibodies (ASCA) are directed against mannan epitopes of *Saccharomyces cerevisiae* and are primarily markers of Crohn's disease but have been reported in some patients with autoimmune hepatitis and other autoimmune liver diseases; their presence is nonspecific and they are not involved in the pathogenesis of AIH [34].

Antibodies to double-stranded DNA have been recognized in AIH for decades and cause homogeneous ANA pattern similar to one seen in systemic lupus erythematosus (hence the former name ‘lupoid hepatitis’). Anti-dsDNA antibodies are present in ∼30% of AIH patients, the frequency is even higher in AIH-PBC overlap syndrome [35]. Anti-histone antibody is frequent in ANA positive AIH patients and the only known specificity in approximately half of these patients. Most of the antibodies are of the IgG class directed to histone H3 [36]. Anti-nucleosome (or chromatin) antibodies are present in 40-70% of AIH patients, more common in men and associated with high IgG level [37,38].

Of known ANCA specificities, anti-lactoferrin and anti-myeloperoxidase antibodies were detectable in 19% and 59% in a Chinese study of 59 patients. Anti-myeloperoxidase was present in PBC and PSC as well, while anti-lactoferrin tested positive only in PSC [39].

#### 2.2.3 Serological subtypes of autoimmune hepatitis based on autoantibodies

Autoimmune hepatitis (AIH) can be classified into serological subtypes based on disease-specific autoantibody profiles, which are mostly mutually exclusive. This categorization was thought to be helpful in diagnosis, clinical characterization, and, in some cases, prognosis, but some studies showed no significant association with clinical parameters and long-term outcomes, especially in adults [40,41]. This is reflected in the guidelines: most documents mention these subtypes but do not incorporate them into recommendations or even discourage their use [[9], [10], [11], [12], [13]].

Type 1 AIH, the most prevalent variant (70–80% of cases), is characterized by antinuclear antibodies (ANA) and/or smooth muscle antibodies (SMA) and is associated with hypergammaglobulinemia. It affects all age groups but is most common in adolescents and adults, with a generally favorable response to immunosuppression, though relapse is frequent after withdrawal [21,22].

Type 2 AIH**,** accounting for ∼10–15% of cases, is typically seen in children or young adults and is defined by anti–liver kidney microsomal type 1 (anti-LKM1) or anti–liver cytosol type 1 (anti-LC1) antibodies. It often presents acutely, with higher risk of severe hepatic injury and a greater likelihood of requiring lifelong maintenance therapy [42,43].

A subset of patients is positive for anti–soluble liver antigen/liver pancreas (anti-SLA/LP) antibodies, which are highly specific for AIH and may be the only serological marker. These patients often display more severe disease and increased relapse rates [22]. Historically referred to as “type 3 AIH,” anti-SLA/LP–positive cases are now classified under type 1 AIH.

#### 2.2.4 Seronegative autoimmune hepatitis

Approximately 5-20% of AIH patients are autoantibody-negative at the time of diagnosis despite having typical clinical and histological features, although definition of ‘seronegative’ is variable [9,[44], [45], [46]]. Antibody titers can be low or undetectable early in the disease**,** particularly in children and in acute severe presentations [13,47]. Conversion to seropositive status can occur later in the disease course improving diagnostic certainty. Repeat testing is recommended when AIH is strongly suspected but initial antibody screening is negative, because some antibodies, especially ANA and SMA, may appear later [21,48,49]. At the same time, later appearance of anti-LKM1 and anti-LC1 is unlikely, sequential analysis of 117 AIH patients for an average of 70 months did not show seroconversion in initially negative cases [50].

Despite repeated testing a part of AIH patients still remain seronegative. Autoantibodies are crucial in the diagnostics of AIH but seronegativity does not rule out the diagnosis. This seronegative gap is reflected in all diagnostic scoring systems of AIH: autoantibody positivity is not mandatory, but a specific value is assigned to it, which – together with other items – makes up the final score. In these cases, the result of histology is essential and provides the final diagnosis of AIH despite autoantibody negativity [9,10,45]. In AIH the histological testing is mandatory in general with few exceptions (e.g., if liver biopsy is contraindicated) since the diagnosis of AIH means a long-term immunosuppressive treatment. According to this, using the simplified AIH scoring system a definitive diagnosis cannot be achieved without a positive liver histology [10]. The initial biopsy and histology might be important at a later time-point of follow-up, too. If response to treatment is suboptimal, the diagnosis of AIH can be questioned but the late biopsy and histology might not be conclusive due to the immunosuppression [11].

#### 2.2.5 Follow-up of AIH specific autoantibody positivity without disease

Studies following individuals with isolated AIH specific autoantibodies are rare, nevertheless serial testing of these individuals can be justified by the growing body of evidence on autoantibodies predicting an autoimmune disease in the asymptomatic phase [[51], [52], [53], [54]]. Haeley et al. followed 251 SMA positive adults for up to 12 years and found that progression from SMA positivity to AIH is rare when ALT is normal, but notable when ALT is raised. Positive predictive value of SMA with raised ALT for AIH was 22%. It should be emphasized that this study included only tubular and glomerular SMA positive cases [55].

### 2.3 Monitoring of appearance of other autoimmune disorders

#### 2.3.1 Overlap/variant syndrome

The prevalence of autoimmune diseases associated with AIH ranges between 20% and 40%, depending on different studies [56]. The most common AIH overlap syndromes are AIH-PBC, AIH-PSC, and AIH-cholestatic syndrome. In AIH-cholestatic syndrome, a cholestatic pattern of liver injury can also be observed, but the serological, histological or cholangiographic features characteristic of PBC and PSC are absent, such as autoimmune cholangitis and anti-mitochondrial antibody (AMA) negative PBC. The overlap between PBC and PSC is extremely rare, with only a limited number of cases documented in the literature [57].

The PBC/AIH and AIH/PBC occur more frequently in middle-aged women and are usually associated with typical cholestatic laboratory features. In all such cases, screening for PBC-specific autoantibodies should be performed — namely mitochondrial antibodies (AMA) specific for M2 antigens, as well as PBC-specific ANA, such as antibodies against gp210 and sp100. Although the specificity and sensitivity of these antibodies in the context of AIH have not been sufficiently studied, and low levels of these autoantibodies may also be detectable in pure AIH, particularly when significant hypergammaglobulinemia is present. Repeated serological testing during follow-up is recommended. PBC variant syndrome (PBC/AIH and/or AIH/PBC) may occur simultaneously, and the two diseases may also develop sequentially. However, primary manifestation of AIH followed by later development of PBC features is more common, although acute flares with an acute AIH-like presentation may also occur in patients with long-standing PBC. Therefore, diagnostic evaluation of these variant conditions may be justified not only at the time of initial diagnosis but also during follow-up. Several expert centers repeat screening of key autoantibodies, as well as immunoglobulin G and M levels, and regularly (annually or biennially) measure aminotransferases and cholestatic enzyme levels [10].

#### 2.3.2 Extrahepatic autoimmunity

Patients with AIH and their first-degree relatives are at increased risk of developing extrahepatic autoimmune diseases [58,59]. At the time of diagnosis, approximately 20% of patients already had such disease, and in 13% of patients new or additional extrahepatic autoimmunity developed within the first 5 years after diagnosis [58]. The most common associated diseases include autoimmune thyroid disease (8%–18%), skin diseases (8%), and inflammatory bowel disease (IBD, 4%–22%) [58] [60] [59]. Celiac disease is also associated with AIH, with a significantly higher prevalence than in the general population (4% vs. 0.4%), and even higher in children (between 11% and 46%) [61] [62] [63]. Based on a large Danish study, the presence of extrahepatic autoimmune diseases affects overall mortality, and patients with more than one extrahepatic autoimmune disease have an even higher mortality rate [58]. These data suggest that regular monitoring of associated diseases and their biomarkers (including autoantibodies) is recommended. Screening of autoimmune thyroid disorders (TSH, anti-thyroid antibodies) and coeliac disease (anti-tissue transglutaminase antibodies) is recommended at least at diagnosis because these conditions are most common and often asymptomatic [10].

### 2.4 Autoantibodies as prognostic markers in autoimmune hepatitis

The prognostic value of ANA and SMA is limited; their titers decrease during immunosuppressive therapy in the majority of AIH-1 patients, however the degree of temporal fluctuation in titers does not predict disease course and outcome [64]. Chen and colleagues, in a systematic review and meta-analysis, found that the presence of anti-SLA increases the likelihood of developing severe liver failure by 3.1-fold [65]. While based on Zachou's study, antibodies against SLA/LP are not able to identify patients with more severe disease or worse survival rates. However, SLA/LP-positive antibodies require lifelong immunosuppression, as they are less likely to achieve corticosteroid withdrawal and have a higher relapse rate after treatment discontinuation. Therefore, in all cases of SLA/LP-positive antibodies, close, long-term monitoring should be recommended after cessation of immunosuppressive therapy [66]. Yüksekyayla and colleagues analyzed and compared clinical characteristics, responses to therapy, and outcomes between anti-SLA/LP positive and negative AIH patients in a prospective study. Their results suggest that anti-SLA/LP positivity does not entail clinically distinct or more severe symptoms in AIH, and that anti-SLA/LP positive patients responded more rapidly to immunosuppressive therapy [67]. Prognostic power of anti-SLA/LP may be overestimated because anti-Ro52 was not tested in all studies and is often present concurrently. Montano-Loza et al. demonstrated that anti-Ro52 alone was associated with cirrhosis development and hepatic death or liver transplantation [27].

The severity of childhood AIH and response to therapy are independent of antibody status; anti-LKM antibody-positive patients show a similar spectrum of disease activity, remission rates, and long-term prognosis as patients with anti-nuclear and/or smooth muscle antibody-positive AIH [68]. Another study also found no difference in outcomes between type 1 and type 2 hepatitis [69].

Evaluation of prognostic role of anti-LC1 is difficult because its association with other antibodies, in a study of 18 patients with isolated positivity outcome was not different from other AIH patients [70].

### 2.5 Monitoring disease activity and autoantibodies

Biochemical markers are essential in follow-up of AIH. Alanine aminotransferase (ALT) and aspartate aminotransferase (AST) are primary markers of hepatocellular injury. Normalization is a key target of therapy; persistent elevation suggests ongoing inflammation or incomplete response. Serum bilirubin and alkaline phosphatase (ALP) are usually less important but useful in detecting cholestatic overlap (e.g., with primary sclerosing cholangitis). International normalized ratio (INR) is used to assess synthetic liver function, especially in advanced disease or suspected acute severe AIH [9,10]. Among immunological markers high IgG level is a hallmark of AIH. Serum IgG typically falls with effective therapy and normalizes in remission [10,71].

Autoantibodies are not recommended as activity markers in general but there is evidence of correlation between their titer and disease activity, though sequential evaluation of antibody levels in AIH patients is limited. Direct comparison with histological activity is rare due to the low number of studies with longitudinal analysis of biopsy samples, usually biochemical tests are used as surrogate markers. It should be noted that these biochemical tests are not entirely reliable measures of disease activity [72].

#### 2.5.1 Antinuclear antibody

Correlation of ANA titer with disease activity is low, in accordance with the guidelines for antinuclear antibody determination which do not recommend ANA testing for monitoring purposes [73,74]. In a study by Gregorio 14 children with AIH were monitored for up to 5 years by ANA, SMA, LKM-1, anti-ASGPR and anti-LSP (liver specific lipoprotein) testing. Titers of all autoantibodies, except ANA were correlated with AST used as biochemical marker of disease activity. Interestingly, IgG antibodies to rubella and tetanus toxoid also correlated with disease activity, while anti-pneumococcal capsular polysaccharide did not. This suggests that T lymphocytes may play an important role in both disease and B cell activity in AIH [75].

#### 2.5.2 Anti-smooth muscle antibody

Smooth muscle antibodies correlate with activity, even disappear and reappear during disease course [76] [77]. Persistently high level of SMA (or anti-actin antibodies) is associated with disease activity [50]. Association of SMA and disease activity is especially true in children, therefore SMA is part of the definition of disease remission [13,78]. Guéguen et al. found a correlation between liver disease activity and double reactivity of anti-F-actin and anti-alpha-actinin [31].

#### 2.5.3 Anti-SLA/LP

Durability of this antibody was reported in 96% of patients who were tested for anti-SLA more than once, change to negative or positive result occurred with equal frequency. Over the course of ten years of monitoring, the likelihood of anti-SLA emerging or disappearing was very low. [27]. In a meta-analysis there was no significant association between anti-SLA and liver histological scores for inflammation, IgG and total bilirubin level but AST enzyme activity was lower in anti-SLA positive patients [65].

#### 2.5.4 Anti-LKM1

Cytochrome P450 2D6 (CYP2D6), the target of LKM-1, was detected on the liver cell membrane, which may show its potential as a pathogenic antibody in AIH [79]. Anti-LKM1 level correlated with serum AST in children with AIH [75]. Another study reported the opposite: anti-LKM1 antibody remained elevated in most patients independently of decreasing ALT levels [80]. Nevertheless, follow-up of anti-LKM1 is also recommended in children with AIH, negative or low titer (<1:10) antibody can be used as a marker of immunological remission [13].

#### 2.5.5 Anti-LC1

Anti-LC1 is often coexistent with anti-LKM1 which impedes the evaluation of correlation with disease activity on its own. In the study by Muratori et al. anti-LC1 disappeared or the concentration decreased in parallel with the ALT level [80]. In another paper, isolated anti-LC-1 antibody level was reported to be associated with biochemical remission in the majority of patients and changed to negative in half of the cases [70].

#### 2.5.6 Anti-asialoglycoprotein receptor antibody

Association between anti-ASGPR antibody and disease activity is contradictory, some studies reported association, others did not [81]. In a study using a novel ELISA, levels correlated with transaminase levels. Moreover, during follow-up elevation of anti-ASGPR preceded liver-transaminase increase [82]. Immunoglobin G, ALT, interleukin-6, interleukin-10 levels were higher, while complement C3 lower in AIH patients with anti-ASGPR positivity compared to those with negative result [83].

#### 2.5.7 Other autoantibodies

Correlation of anti-alpha-actinin and anti-ssDNA double positivity and both clinical and histological activity of the disease was reported in untreated AIH-1 patients [84]. Yokokawa et al. found that anti-nucleosome antibody levels were significantly lower during remission compared to levels during flares [37]. Chromatin antibodies were detected more frequently during active than inactive disease and disappeared in 42% of patients tested repeatedly [85]. In a human protein microarray analysis study, antibodies against docking protein 2 correlated with serum IgG and histological activity [86].

### 2.6 Role of autoantibodies in the treatment of autoimmune hepatitis

Treatment goal in AIH is to achieve complete biochemical, clinical, and histological remission. Most patients receive long-term, often lifelong, immunosuppressive therapy. Treatment usually starts with steroid induction therapy followed by maintenance using a steroid sparing agent. Treatment choice is based on disease activity and severity, histological activity, fibrosis stage, contraindications to steroids or azathioprine, treatment tolerance and side-effects [87]. Current guidelines do not recommend autoantibodies to guide choice of therapy. Prognostic autoantibody markers can be considered as factors in modulating therapy, especially in the decision on treatment cessation. E.g., anti-SLA/LP positive patients may need lifelong immunosuppression [10,66].

Connection between autoantibodies and initial treatment response is very weak. Czaja et al. found that SMA and ANA is frequently lost but disappearance does not predict treatment outcome [76]. Some data exists that anti-LKM may be suitable for monitoring response to therapy, but criteria of assessing therapy response are variable [[75], [77], [80]]. Pape et al. redefined treatment response and endpoint in AIH in a recent study by the International Autoimmune Hepatitis Group (IAIHG). No autoantibody test was utilized in these new definitions of complete biochemical response, insufficient response, non-response, remission and intolerance to treatment. Moreover, apart from one clinical trial using changes in ANA/SMA titer, antibody testing was not mentioned among published definitions for endpoints in AIH treatment between 1970 and 2015 [71].

SMA positivity and titer was associated with higher relapse rate in a small Japanese cohort [88]. According to a meta-analysis, anti-SLA positive patients have a more than two-fold risk of relapse after IST withdrawal (OR: 2,24) compared to anti-SLA negative population [65]. In a longitudinal study covering 20 years of follow-up of children with AIH, relapse rate after immunosuppression withdrawal was higher in anti-LKM1 positive cases as compared to ANA/SMA positive ones [89]. Anti-LC1 antibodies shows a significant decrease (>50%) or disappearance during remission and rises again at relapse [80]. Titer of isolated anti-LC1 increased in 2 out of 18 patients during relapse [70]. Anti-ASGP-R antibody levels may decrease during immunosuppression and increase in case of disease relapse [90].

In the general, autoantibodies are unreliable markers of disease relapse in AIH, patients should be monitored by measuring aminotransferase levels and IgG [10].

### 2.7 Autoantibodies in the monitoring of complications

Autoantibodies play a central role in the diagnosis of AIH. However, their value for disease monitoring is limited, particularly with regard to predicting complications. Historical divisions into AIH-1 (ANA/SMA), AIH-2 (anti-LKM1/anti-LC1), and AIH-3 (anti-SLA/LP) were proposed, but growing evidence indicates that autoantibody profiles do not predict long-term outcomes. Large observational and cohort studies have shown comparable clinical, biochemical, histological, and prognostic features across serological subgroups, including patients with anti-LKM1 or anti-SLA/LP antibodies. Apparent prognostic associations previously attributed to specific autoantibodies, such as anti-SLA/LP, may instead reflect coexisting markers (antibodies to Ro52) rather than a distinct disease phenotype. Consequently, routine subclassification of adult AIH according to autoantibody profile is not recommended [10]. The development of complications is primarily assessed using biochemical parameters and histological findings. Nevertheless, autoantibodies may have an indirect role in certain clinical contexts.

#### 2.7.1 Acute liver failure

Acute liver failure (ALF) represents a severe complication of AIH. Autoantibodies may facilitate differential diagnosis but are not suitable for predicting or monitoring this complication.

Acute-onset AIH poses significant diagnostic difficulties, as its serological and histopathological features may differ from those observed in classical disease presentations. In this setting, the absence of ANA and ASMA has been documented in approximately 11–17% of patients [[91], [92], [93], [94]]. Moreover, autoantibodies lack disease specificity, as evidenced by the detection of ANA and ASMA in 31.3% and 18.0% of patients with ALF of non-autoimmune etiology, respectively. [94]. Furthermore, in acute presentations, autoantibody titers are often reduced or may be transiently negative; therefore, clinical status and laboratory parameters are the primary determinants of diagnosis and management [95].

Earlier reports suggested that certain autoantibody profiles in AIH are associated with a more severe disease course and therefore warrant closer clinical monitoring. AIH-2, characterized by anti-LKM-1 autoantibodies, is predominantly seen in younger patients and is more frequently associated with acute or fulminant presentations, including ALF, than type 1 AIH [96]. Multiple studies have indicated that anti-SLA/LP autoantibody positivity in AIH patients is associated with a more aggressive disease course as well [97]. Furthermore according to Czaja et al. anti-actin positive adult AIH-1 patients tend to develop liver failure more often and respond less effectively to corticosteroids than seronegative patients [98].

#### 2.7.2 Fibrosis, cirrhosis

Fibrosis assessment relies on liver biopsy or non-invasive methods such as transient elastography, or serum-based indices (e.g., FIB-4 score) in AIH [99]. The utility of autoantibody levels as a stand-alone predictor of liver fibrosis or cirrhosis in AIH is limited due to variability in titer levels and reactivity over time. The findings are inconsistent in other liver diseases as well. For example in metabolic dysfunction-associated steatotic liver disease (MASLD), ANA and SMA status may serve as a potential predictive marker for advanced liver disease, including cirrhosis [100]. However, the presence of antibodies was not associated with fibrosis in another study [101].

A noteworthy observation that certain autoantibody profiles, for example anti-SLA/LP positivity, have been associated with a more aggressive disease course and may therefore carry prognostic significance regarding fibrosis or cirrhosis. Anti-LC1 positivity is also associated with liver inflammation and rapid progression to cirrhosis [102].

#### 2.7.3 Monitoring hepatocellular carcinoma in autoimmune hepatitis

According to a multicentric study of patients included in the International Autoimmune Hepatitis Group Retrospective Registry, incidence of hepatocellular carcinoma is low, even if cirrhosis is present. ANA, SMA and anti-LKM1 antibodies were included as potential predictors of carcinoma development but not found significant [103]. In Asian patients the incidence was higher but predictors were cirrhosis and hepatic decompensation, no autoantibodies were found important [104].

#### 2.7.4 Recurrence of autoimmune hepatitis after liver transplantation

Recurrence of AIH following liver transplantation occurs in approximately 36–68% of cases after 5 years [105]. Based on available evidence, the autoantibody profile does not appear to meaningfully influence the risk of disease recurrence after liver transplantation. However, high titers of autoantibodies at the time of liver transplantation have been identified as significant risk factors for recurrent AIH [106].

Comparative studies have shown no significant differences in recurrence rates between patients with type 1 (ANA/SMA-positive) and type 2 (anti-LKM1-positive) autoimmune hepatitis [107]. Importantly, conventional autoantibodies may also be detected in graft rejection, limiting their diagnostic specificity for recurrent disease [108].

Conventional serological markers (ANA, SMA, and anti-LKM1), often reappear when autoimmune hepatitis recurs [[109], [110], [111]]. Anti-LKM1 antibodies are less frequently observed in recurrent disease - likely due to the rarity of type 2 autoimmune hepatitis in adult transplant populations [112] and possibly a lower recurrence tendency [111].

Overall, autoantibody status alone is not a reliable predictor of autoimmune hepatitis recurrence after transplantation**.** Monitoring remains primarily based on biochemical and histological evaluation; however, autoantibody positivity may raise clinical suspicion and prompt further investigation, including liver biopsy.

### 2.8 Autoantibodies in monitoring autoimmune hepatitis in special clinical contexts and risk populations

#### 2.8.1 Pregnancy

AIH is more common in women, particularly those of childbearing age. In diagnosed AIH patients pregnancy-related immune modulation may alter disease activity, with flares most frequently observed in the postpartum period rather than during gestation [113].

Although autoantibody titers are not reliable markers of disease activity during pregnancy, the presence of specific autoantibodies may carry prognostic significance for pregnancy outcome. In pregnant patients, antibodies against SLA/LP and Ro/SSA have been shown to be strongly associated with otherwise unexplained adverse pregnancy outcomes [114]. This suggests that, while serology has limited value for monitoring disease course, certain autoantibodies may contribute to risk stratification rather than disease activity assessment. Accordingly, the identification of these antibodies may justify closer maternal and fetal surveillance throughout pregnancy and the postpartum period.

However, despite their relevance in selected high-risk patients, routine screening for liver autoantibodies is not recommended during pregnancy, as de novo autoimmune liver disease is uncommon.

#### 2.8.2 De novo autoimmune hepatitis after liver transplantation

De novo AIH develops after liver transplantation performed for non-autoimmune indications and affects approximately 1–7% of recipients. The most common autoantibodies detected are ANA and SMA, either alone or in combination. Anti-LKM have been described mainly in pediatric cases, while antibodies to gastric parietal cells and liver cytosol have also been reported. Importantly, de novo AIH may occur in the absence of detectable autoantibodies [115].

A disease-specific nonstandard serological marker has been identified in adult de novo AIH: an atypical anti–liver/kidney cytosolic antibody targeting glutathione S-transferase T1 (GSTT1) [116]. Anti-GSTT1 antibodies can be present with the conventional markers or are the sole serological finding and occur predominantly in GSTT1-negative recipients of GSTT1-positive grafts [33,117,117].

The development of autoantibodies after liver transplantation is a significant risk factor for de novo AIH. In pediatric recipients, post-transplant autoantibody production - most commonly SMA - has been associated with an increased risk of de novo AIH and chronic rejection, as well as progressive fibrosis [118,119]. Similar associations have been observed in adults, particularly in the context of donor–recipient GSTT1 mismatch, where anti-GSTT1 antibodies strongly predict de novo AIH development [117,120]. Immunosuppressive regimen also influences risk, as tacrolimus therapy is associated with lower anti-GSTT1 production and reduced incidence of de novo AIH compared with cyclosporine [121]. Collectively, these findings support the use of post-transplant autoantibody surveillance for early identification of patients at risk for de novo AIH, but autoimmune serology is not recommended as a routine screening tool in asymptomatic patients with stable graft function.

#### 2.8.3 Drug induced autoimmune hepatitis

Drug-induced autoimmune-like hepatitis (DIAIH) is an important differential diagnostic problem in patients presenting with autoimmune features during liver injury, most frequently associated with minocycline, nitrofurantoin, hydralazine, methyldopa, statins, fenofibrate, alpha and beta interferon, infliximab and etanercept [122]. Autoimmune serology is commonly positive in this context: according to a multicenter retrospective study, up to 72% of cases exhibit ANA, 42% SMA, 2% LKM and 2% SLA/LP autoantibodies [123]. With clinical improvement, both autoantibody titers and immunoglobulin levels typically decline, and in many cases autoantibodies disappear altogether; however, low-level autoantibody positivity may persist over prolonged follow-up [122], underscoring that serology in DIAIH reflects immune activation secondary to drug exposure rather than a sustained idiopathic autoimmune process.

Interestingly, a study published by Chalasani et al. suggests that immunoglobulin class–specific autoantibody profiles may help distinguish DIAIH from de novo AIH [124]. While de novo AIH is characterized by a mixed IgG and IgM autoantibody response, DIAIH appears to be predominantly associated with IgM autoantibodies. In exploratory modeling analyses, a panel of five IgG autoantibodies (directed against CENP-B, chromatin, mitochondrial antigen, myosin, and nucleosome) discriminated de novo AIH from DIAIH with high accuracy (AUC 0.88), whereas a distinct IgM autoantibody signature (dsDNA, SCL-70, ssDNA, U1-snRNP-B/B’) was predictive of DIAIH compared with non-autoimmune DILI (AUC 0.87). Although not yet applicable to routine clinical practice, these findings highlight the potential of advanced serological profiling to refine differential diagnosis in immune-mediated liver injury.

Consequently, serial autoantibody monitoring has limited utility in tracking disease activity or guiding long-term management; decisions are better informed by biochemistry parameters, IgG levels, and clinical response to drug cessation or immunosuppression**.**

#### 2.8.4 Autoimmune hepatitis and immune checkpoint inhibitor therapy

By enhancing T-cell–mediated immune responses, immune checkpoint inhibitors (ICIs) such as CTLA-4, PD-1, and PD-L1 inhibitors may disrupt hepatic immune tolerance and trigger immune-mediated liver injury that can resemble classical AIH.

In patients receiving ICIs, routine baseline screening for liver-related autoantibodies is not recommended. According to current international guidelines (ESMO, SITC, NCCN, ASCO), liver autoantibody serology should be performed selectively in the setting of suspected immune-related hepatitis, particularly in patients who develop grade ≥2 toxicity. In this context, recommended testing includes ANA, ASMA, LKM, and SLA/LP antibodies [[125], [126], [127], [128]].

Autoantibodies traditionally associated with idiopathic AIH such as ANA and SMA are generally not present in checkpoint inhibitor induced liver injury (ChILI). In a French multicenter cohort of 117 patients, ANA positivity was observed in 7 cases of cholestatic hepatitis (6%), 4 cases of mixed hepatitis (3.4%), and 5 cases of hepatocellular hepatitis (4.3%), while SMA positivity was rare, occurring in only 1 patient with mixed hepatitis (0.9%) and 5 patients with hepatocellular hepatitis (4.3%) [129].

Autoantibody assessment is primarily intended to support the differential diagnosis, exclude underlying or overlapping autoimmune hepatitis, and guide further diagnostic and management decisions, rather than to establish the diagnosis of immune-related hepatitis itself.

## 3 Conclusions

Autoantibodies are immunological hallmark of autoimmune hepatitis and some of them, especially antinuclear antibodies, smooth muscle antibodies, anti–liver kidney microsomal type 1, anti-liver cytosolic antigen 1 or anti–soluble liver antigen are indispensable for diagnosis. However, their role in disease follow-up and monitoring is substantially more nuanced and limited (Table 2). The available evidence indicates that, for most AIH-specific and associated autoantibodies, antibody status is largely stable over time and titer does not consistently parallel biochemical activity, histological inflammation or treatment response. Importantly, these associations are heterogeneous across studies, probably due to differences between populations and variability of methods and reagents used in autoantibody testing. Consequently, routine serial measurement of conventional AIH autoantibodies is not supported as a reliable surrogate marker of disease activity or remission and do not need to be monitored regularly unless a significant change in the clinical phenotype does appear.

**Table 2:** Role of autoantibodies in the diagnostics, prognostics and follow-up of patients with autoimmune hepatitis.

Antibody	Diagnosis	Prognosis	Disease activity	Treatment
ANA	**+++**	+/−	**-**	**+**
SMA	**+++**	+/−	**++**	**+**
Anti-SLA/LP	**++**	**+++**	**-**	**+++**
Anti-Ro52/TRIM21	**-**	**++**	**-**	**-**
Anti-LKM1	**+++**	**+**	**+**	**+**
Anti-LC1	**+++**	**+**	**+**	**+**
ANCA	**++**	**-**	**-**	**-**
Anti-ASGPR	**+**	**-**	**+**	**-**

ANA: Antinuclear antibody; SMA: smooth muscle antibody: SLA/LP: soluble liver antigen/liver pancreas; TRIM21: tripartite motif-containing protein 21; LKM1: liver kidney microsomal type 1; LC1: liver cytosol type 1; ANCA: anti-neutrophil cytoplasmic antibody; ASGPR: asialoglycoprotein receptor.

+++: included in the diagnostic scoring systems/guidelines; ++: clear evidence; +: some supporting data are available; +/−: conflicting data; -: no correlation.

In contrast to autoantibody profiles, conventional biochemical markers - especially serum transaminases and immunoglobulin G levels - remain the cornerstone of laboratory monitoring of AIH. Autoantibodies should therefore be interpreted as complementary rather than primary follow-up markers, contributing to risk stratification and phenotypic characterization rather than disease monitoring.

Future research should focus on standardization and harmonization of autoantibody tests to ensure comparability of results. Critical parameters of testing methods should be identified such as initial dilution of sample, source of autoantigen, conservation of antigen conformation, fine epitope specificity of autoantibody etc. Indirect immunofluorescence tests remain mandatory in AIH diagnostics, with the consequent need for alignment of pattern definitions and names, education and quality control of pattern recognition. The emergence and spread of computer-aided or fully automated evaluation of fluorescence patterns in routine medical laboratories, especially those using artificial intelligence, presents a challenge for controlling variability of testing.

Long-term follow-up data of AIH antibody markers are limited, multicentric, longitudinal studies are needed to detect weaker associations, especially in case of autoantibodies with low prevalence. This endeavor may clarify whether specific autoantibody patterns, combinations, or isotypes can meaningfully enhance individualized follow-up strategies in autoimmune hepatitis.

## CRediT authorship contribution statement

**Gábor Nagy:** Writing – review & editing, Writing – original draft, Visualization, Supervision, Methodology, Investigation. **Dóra Bencze:** Writing – original draft, Investigation. **Sarolta Demeter:** Writing – original draft, Investigation. **Krisztina Pénzes-Daku:** Writing – original draft, Investigation. **Lilla Szabó:** Writing – original draft, Investigation. **Beáta Tóth:** Writing – original draft, Investigation. **Róza Földesi:** Writing – original draft, Investigation. **Mária Papp:** Writing – review & editing, Conceptualization. **Péter Antal-Szalmás:** Writing – review & editing, Writing – original draft, Visualization, Methodology, Investigation, Conceptualization.

## Declaration of generative AI and AI-assisted technologies in the manuscript preparation process

During the preparation of this work, the authors used ChatGPT Plus (model: GPT 5) in parallel with a traditional keyword- and search term-based approach in order to support literature search on the internet. After using this tool/service, the authors checked the original document the AI referred to in its answers to specific autoantibody related prompts, reviewed and edited the content as needed, and take full responsibility for the content of the published article.

## Funding

The publication costs of the article were covered by the Science support program of the 10.13039/501100009232University of Debrecen.

## Declaration of competing interest

The authors declare that they have no known competing financial interests or personal relationships that could have appeared to influence the work reported in this paper.

## Data Availability

No data was used for the research described in the article.
